# Pelvic Myxoid Leiomyoma Mass between Vagina and Rectum

**DOI:** 10.1155/2016/3479132

**Published:** 2016-06-26

**Authors:** Omar AlShalabi, Fadi Obaied Alahmar, Hazem Aljasem, Bayan Alsaid, Abdulghani AlShalabi

**Affiliations:** Department of General Surgery, Al-Assad University Hospital, Faculty of Medicine, 10769 Damascus, Syria

## Abstract

Leiomyomas are the most common pelvic tumors in women. About 20–30% of women older than 35 are affected. Rare conditions of leiomyomas have extrauterine locations. Myxoid degeneration is a rare type of leiomyoma degeneration. We report a case of solid-cystic myxoid leiomyoma in a 53-year-old woman complained of constipation, urinary hesitation, and malodorous vaginal discharge with palpable 17 × 12 cm mass between vagina and rectum. Regarding the inferior location of the mass, a perineal approach was used to enucleate it. This rare location has not been mentioned before. The woman was finally diagnosed by pathologists which was myxoid leiomyoma.

## 1. Introduction

Leiomyomas are the most common benign tumor in women [1]. In general, leiomyomas are rubbery solid tumors, but infrequently they may undergo myxoid degeneration. The uterus is the most common location [1], but rare cases have been reported in cervix [2], vaginal canal [3], broad ligament [4], and ovaries [5]. Most leiomyomas are asymptomatic and are diagnosed incidentally. Surgical resection is a part of multimodality treatment. Here, we report a case of myxoid leiomyoma in a 53-year-old woman with pelvic rare location between vagina and rectum; the mass was enucleated by transperineal incision. To our knowledge, this location was not reported before.

## 2. Case Presentation

A 53-year-old woman with no previous medical history was admitted to our center complaining of malodorous vaginal discharge and sever constipation. Her symptoms began a year ago; the patient suffered from moderate constipation and urinary hesitancy. Her symptoms developed to severe constipation, urinary hesitancy, and malodorous vaginal discharge with inability to defecate unless in standing position.

Physical examination and digital rectal exam combined with bimanual transvaginal exam revealed a solid rubbery mass about (12 × 10 cm) between the posterior wall of vagina and anterior wall of the rectum with no obvious limits. The examination under general anesthesia demonstrated the same findings.

Laboratory findings were within normal limits, except for a mild leukocytosis (12000 cells per mm^3^). Vaginal secretions were sent to analysis and revealed fibrin, erythrocytes, and very rare benign endometrial elements.

**Figure 1:**
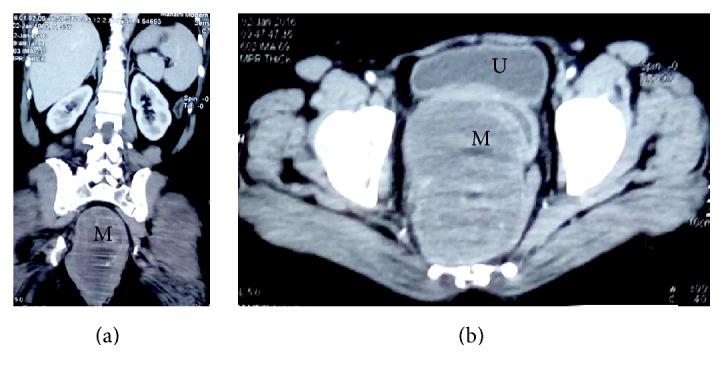
Contrast enhanced computed tomography (CT). (a) Frontal section showing the extension of the mass (M) from the sacrum to the perineum. (b) Transverse section showing the mass (M) compressing the bladder (U) and vaginal canal anteriorly and the rectum posteriorly.

Radiological study with echography and contrast enhanced computed tomography (CT) showed a (17 × 12 × 10 cm) solid-cystic mass extending from sacrum posteriorly to pubic symphysis anteriorly and to perineal skin inferiorly, with mass effect (Figure 1). Sigmoidoscopy showed a mass pressing the anterior wall of the rectum with no mucosal abnormalities.

**Figure 2:**
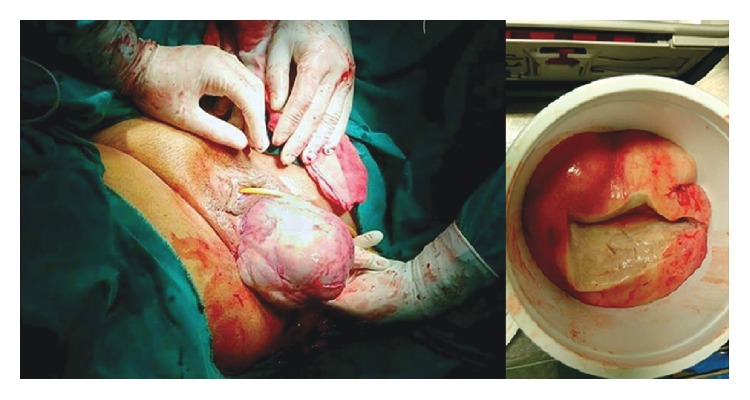
The enucleation process through the transperineal incision and gross appearance of the mass.

Upon laparotomy, a lithotomy position was used, and an abdominal approach was established. The uterine was found enlarged with no other abnormalities. The peritoneum of rectouterine pouch was incised and the big mass was found with no connection to the vagina or the rectum. A perineal incision was made and the mass was enucleated en bloc (Figure 2) without any damage to the surrounding structures, vagina and rectum. We used a corrugated rubber drain which was drone 24 hours later with no complications. The patient was discharged 72 hours after surgery with relief of constipation and urinary hesitancy. She visited the surgical clinic 3 months later with no urologic, gastroenterological, nor gynecologic complications and no recurrence.

Macroscopically, the mass measuring 15 × 15 × 6.5 cm, having well demarcated borders, cut surface revealed a myxoid appearance with occasional nodules of white fasciculated tissue.

**Figure 3:**
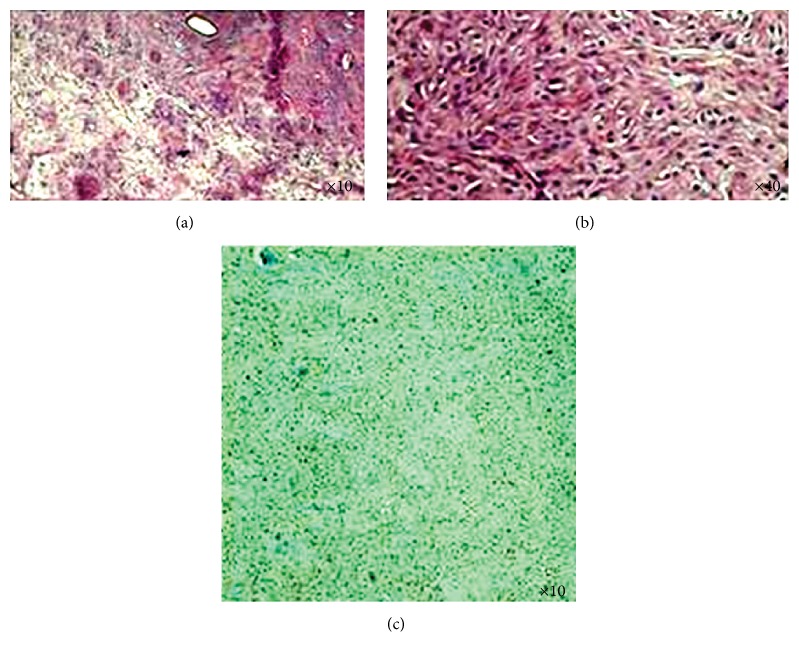
(a) Microscopic aspect (×10) showing the bundles of spindle within edematous myxoid stroma. (b) Microscopic aspect (×40) showing the bundles of spindle cells having elongated bland nuclei. (c) Microscopic aspect (×10) showing the positive nuclear response on estrogen receptor.

Microscopically, the nodules are composed of fascicles and bundles of spindle cells having elongated bland nuclei; the remaining tissue showed thick-walled blood vessels within edematous myxoid stroma. Neither necrosis nor irregular mitotic activity could be seen (Figure 3(a)).

Immunohistochemistry revealed nuclear positive response on estrogen receptor (Figure 3(c)) and a negative result on HMB45 (human melanoma black 45) and Ki67. Actin stained the blood vessels walls.

Malignancy can be excluded and the final diagnosis is consistent with myxoid leiomyoma.

## 3. Discussion

Leiomyomas are the most common pelvic tumors in women [6, 7]. They are benign monoclonal tumors arising from the smooth muscle cells; they arise usually from the uterus, but rare cases have been reported in cervix, vaginal canal, broad ligament, and ovaries [8]. Some reports mentioned unusual growth pattern of leiomyomas like diffuse peritoneal leiomyomatosis, intravenous leiomyomatosis, benign metastasizing leiomyomas, retroperitoneal leiomyomas, and parasitic leiomyomas [9]. According to many documents, it is still unclear if these lesions represent metastatic or synchronous primary lesions or whether they arise from the hormonally sensitive smooth muscle [9]. Some studies suggest that these tumors are independent soft tissue tumors rather than parasitic leiomyomas of the uterus [10]. Others suggest that these tumors can arise anywhere in the body since they probably arise from smooth muscle cells including those in blood vessels [11]. Other authors explained the rare cases of disseminated peritoneal leiomyomas happing in men with no excess hormones, to the increase responsiveness of tumor cells to normal hormone levels [9].

Leiomyomas are usually asymptomatic and discovered through routine ultrasound. Some patients present with mass effect symptoms such as hydroureteronephrosis in retroperitoneal masses, postcoital bleeding in cervical masses, constipation, and urinary hesitancy. In our case, a malodorous vaginal discharge was reported due to leiomyoma's position between vagina and rectum. Since malignancy is more common in retroperitoneal smooth muscle, radiologic study (CT or magnetic resonance imaging (MRI)) is mandatory to evaluate the mass and its relationship to the adjacent structures and blood vessels [11]. Although radiologic study is important, no test is highly sensitive or specific to give a conclusive decision to rule out malignancy, which is done with histopathological examination [11].

All previous reports mentioned that the laparotomy and the laparoscopic surgery through the abdomen are the best ways to resect these tumors. In this case, the abdominal approach was not able to help reach the mass and enable its resection. Transperineal incision allowed the enucleation of the mass.

The histopathologic application could not definitely confirm the origin of the tumor, whether it arose from the genital tract or from the tissue in the retroperitoneum. The rectum, vaginal canal, and Denonvillier fascia (since it contains smooth muscle cells and blood vessels [12]) can all be possible origins of the mass. Immunohistochemistry is the final step in confirming the results and excluding malignancy. The positive response of the actin and estrogen receptors confirms that the tumor has a smooth muscle component and suggests the possibility of a genital tract origin. Absence of necrosis, with Ki 67 negative/low rates, helps exclude leiomyosarcoma; HMB45 negative result helps exclude angiomyolipoma.

## 4. Conclusion

Leiomyomas are benign tumors of smooth muscle. Extrauterine tumors are rare manifestation and can be found anywhere in the body. The resection of the pelvic low tumors may occur through transperineal incision. Malignancies should always be ruled out in retroperitoneal leiomyomas.
